# Translation, cross‐cultural adaptation and validation of the Pain Catastrophizing Scale (PCS) into Bengali in patients with chronic non‐malignant musculoskeletal pain

**DOI:** 10.1111/1756-185X.13954

**Published:** 2020-08-30

**Authors:** Muhammad Shoaib Momen Majumder, Shamim Ahmed, Nahiduzzamane Shazzad, A. T. M. Tanveer Hasan, Syed Atiqul Haq, Johannes J. Rasker

**Affiliations:** ^1^ Department of Rheumatology Bangabandhu Sheikh Mujib Medical University (BSMMU) Dhaka Bangladesh; ^2^ Department of Rheumatology Enam Medical College Savar, Dhaka Bangladesh; ^3^ Faculty of Behavioral, Management and Social sciences Department Psychology, Health and Technology University of Twente Enschede The Netherlands

**Keywords:** Bengali, chronic non‐malignant musculoskeletal pain, cross‐cultural adaptation, Pain Catastrophizing Scale

## Abstract

**Aim:**

To develop a culturally adapted and validated Bengali Pain Catastrophizing Scale (BePCS).

**Methods:**

The English PCS was translated, adapted and back‐translated into and from Bengali, pre‐tested by 30 adult patients with chronic non‐malignant musculoskeletal pain. The BePCS was administered twice with 14 days interval to 90 patients. Convergent validity was measured by comparing the BePCS score with scores of the domains physical functioning and mental health of the Bengali Short Form 36, through Spearman's correlation coefficient. Test‐retest reliability was assessed by intraclass correlation coefficient (ICC) and Spearman's rank correlation coefficient and internal consistency by Cronbach's alpha. Content validity was assessed by index for content validity (ICV) and floor and ceiling effects.

**Results:**

The BePCS was well accepted by the patients in the pre‐test. The content validity was excellent, both item ICV and scale ICV were 1. Construct validity: the convergent validity was −0.424 for physical functioning and −0.413 for mental health, indicating a moderate negative correlation. Total BePCS score showed excellent internal consistency with a mean Cronbach's *α* = 0.92. Internal consistency for subscales rumination, magnification and helplessness, were Cronbach's *α* 0.903, 0.72 and 0.872 respectively. The test‐retest reliability of total BePCS was 0.78 (*P* < .001) and for the subscales rumination 0.872 (*P* < .001), magnification 797 (*P* < .001) and helplessness 0.927 (*P* < .001), showing excellent test‐retest reliability.

**Conclusions:**

The interviewer‐administered BePCS appears to be an acceptable, reliable and valid instrument for measuring health‐related quality of life in Bengali speaking patients with chronic non‐malignant musculoskeletal pain. Further evaluation in the general population and in different medical conditions should be done.

## 1 INTRODUCTION

Pain as a symptom is now considered the 5th vital sign^1^; it accounts for approximately 80% of physician visits and for an estimated US$ 100 billion annually regarding cost of healthcare and loss of productivity.^2^ Chronic non‐specific musculoskeletal pain is a burden for patients. It is associated with high socio‐economic costs^3^, ^4^, ^5^ and significantly affects the psychosocial status of affected people as well as their families and carers.^6^

Chronic pain has complex underlying pathophysiology, and is determined by multiple psychological, social and biological factors. One of these factors is pain catastrophizing, characterized by patients magnifying their feelings about painful situations and continually thinking about these situations.^7^ Catastrophizing also involves feelings of helplessness and rumination about pain. Pain catastrophizing is related to multiple health outcomes like pain intensity, interference of pain with patients’ lives, physical disability and mental well‐being.^8^ Pain catastrophizing causes a negative mental setting to bear actual or anticipated pain.^9^ Pain feeling has been found to increase from 7% to 33% in pain ratings, depending on the extent of catastrophizing.^10^ Catastrophizing plays an important role in pain chronicity and has a positive correlation with pain intensity and disability.^11^ It not only causes an increased perception of pain and emotional stress, but also prolongs pain episodes and catastrophizing is a significant predictor of the severity of pain, and of the ways in which people cope with pain.^12^ ^,^ ^13^ Catastrophizing thus influences various substantial pain‐related outcomes including: greater pain intensity and chronicity, depression, anxiety, pain‐related disability and analgesic use.^14^ Pain catastrophizing has been associated with poor pain treatment response in patients with chronic pain.^9^ Previous studies reveal that if pain catastrophizing diminishes, pain intensity, disability and chronic conditions would decrease.^15^ It appeared possible to modify pain catastrophizing in patients undergoing surgery.^16^ In psychological research it is postulated that pain catastrophizers may enact pain behaviors in order to receive support or empathy from their social environment.^17^ It has been shown that higher levels of catastrophizing pain behavior were associated with a more intense inference of pain by the observers, which may lead to over‐cautious treatment decisions by those who take care of these patients.^17^, ^18^

The Pain Catastrophizing Scale (PCS) was developed in 1995 by Sullivan et al to measure the individual degree of pain catastrophizing. The PCS is a multidimensional questionnaire, consisting of 3 subscales: helplessness, magnification, and rumination. The English version of the PCS has been investigated extensively, and its psychometric properties are good.^19^, ^20^ The psychometric properties of the questionnaire have been confirmed at least for 10 other languages, including German, Brazilian, Chinese, Portuguese, and Arabic.^21^, ^22^, ^23^, ^24^ There are more than 164 million people in Bangladesh^25^ and about 265 million Bengali speaking people worldwide and it is the 7th language according to population.^26^

A culturally adapted and validated Bengali version of the PCS for the people of Bangladesh is not yet available. The purpose of this study is the translation of the PCS into Bengali, cultural adaptation of the Bengali version and to test its validity and reliability in adult patients with chronic non‐malignant musculoskeletal pain.

## 2 MATERIALS AND METHODS

### 2.1 Patients

The study has been conducted in the Department of Rheumatology (inpatient and outpatient), of the Bangabandhu Sheikh Mujib Medical University.

Consecutive adult female/male patients between 18 and 70 years of age, who visited the rheumatology outpatient and inpatient departments between September 2015 to August 2016, who suffered from chronic non‐malignant musculoskeletal pain (pain persisting ≥ 6 weeks) (10) at the spine or any part of the body and who consented to participate were enrolled in this study. Excluded were severely ill patients, patients with communication problems, patients who had a history of malignant disorders, those who suffered from alcohol or substance abuse and those who had acute pain or needed urgent surgery or other interventions. Substance/alcohol abuse was identified by taking histories and defined as: alcohol/substance used in amounts which are harmful to the individual or others.

The sample size of the study was 95 patients, as calculated by Study Size 3.0, a validated statistical software developed by Creostat HB35 HB in Sweden.^27^ Our expected intraclass correlation coefficient (ICC) for the assessment of test‐retest reliability was 0.9 and the minimal acceptable ICC was 0.7. So using a two‐sided test with *β* = 0.2 (80% power) and *α* = 0.05, the sample size required was 22.873. Thus for the assessment of the test‐retest reliability of the questionnaire and considering drop‐out of some patients during retesting, a sample size of 32 was considered to be sufficient. These 32 patients were collected by simple random sampling from the 95 patients who were enrolled for the test.

### 2.2 The PCS

The PCS was developed in 1995 at the University Center for Research on Pain and Disability of the McGill University of Canada by Michael JL Sullivan, in order to facilitate research on the mechanisms by which catastrophizing develops and its impact on pain experience.^28^ The PCS is a 13‐item instrument with 5‐point scales with the endpoints (0) not at all and (4) all the time. The PCS yields a total score and 3 subscale scores assessing: rumination, magnification and helplessness. It can be scored by summing all of the ratings for each subscale (range, 6‐item helplessness 0‐24; 3‐item magnification 0‐12; 4‐item rumination 0‐16 points) or by the total score of its 13 items (range 0‐52 points) with higher scores representing greater pain catastrophizing. Patients having a PCS score of more than 30 represent a clinically relevant level of catastrophizing and are considered at high risk for the development of chronic pain/disability, and a score of >30 is an indication for considering psychological intervention.

### 2.3 Translation procedure

For translation and validation of the Bengali version of PCS, we obtained permission from the original author (MJ Sullivan). For translation and cultural adaptation of the English PCS into Bengali, we followed the recommendations by Beaton et al^29^ Forward translation was carried out by 2 translators whose mother tongue is Bengali. One of the translators was the first author (MSMM), and the other was a Bengali teacher working in Dhaka University who was not apprised of the translation background. Comprising both translations, a synthesized form of the Bengali version was formed. Two English linguistic professionals – one from the Department of English, Dhaka University, another from a local college of Dhaka – translated the synthesized Bengali version of PCS into English (back‐translation). An expert committee composed of 5 persons – a language professional, 3 rheumatologists and 1 statistician – reviewed and compared all the translations and the original English PCS. They verified the semantic, idiomatic, experiential and conceptual equivalence between the English and Bengali versions; a consensus was reached to form 2 sets of the prefinal Bengali version of the questionnaire. The 2 questionnaires differed in the wording of some of the items.

### 2.4 Testing of prefinal version

The 2 prefinal Bengali versions of the PCS were tested in a sample of 30 adult patients with chronic non‐malignant musculoskeletal pain. Each subject completing the questionnaires was interviewed to find out what he or she thought was meant by each questionnaire item, and about the response they gave, and whether they had any further suggestions. If a participant was able to understand both of the translations of the same item, he or she was asked which translation (in the prefinal version ‐ 1/2) he or she would prefer. Based on the response of these participants, the adapted version was prepared.

The adapted version was administered twice with 14 days interval to 90 Bangladeshi patients who were suffering from chronic non‐malignant musculoskeletal pain.

For measuring the physical functioning and mental health, these domains of the Bengali version of the Short Form‐36 (SF‐36) were applied.^30^

### 2.5 Questionnaire administration

The questionnaire was used as a self‐administered one for literate participants and an interviewer‐administered one in case of illiterate participants. The literate participants were allowed to read the questionnaire themselves and give the replies as per their own understanding. In case of illiterate participants, the interviewer read the questionnaire in a clearly audible voice, without giving explanation. The responses were recorded by the interviewer.

### 2.6 Statistical analysis

All data were assessed using SPSS 22.0 (SPSS Inc). All tests were 2‐tailed and conducted at a 5% level of significance. There were no missing data for any items. Both the content validity and construct validity were assessed. Reliability was assessed through three ways: internal consistency, test‐retest reliability and item to scale correlation. The internal consistency was measured using Cronbach's alpha. The internal consistency was considered acceptable when Cronbach's alpha was equal to or exceeded 0.70.^31^ The item to scale correlation was assessed using Spearman's rank correlation (rho) between scale and their constituent items, taking a value of rho ≥ 0.40 as acceptable.^32^ Test‐retest reliability was assessed using ICC. An ICC between 0.60 and 0.74 was considered good, between 0.75 and 1.00 was excellent and considered acceptable for test‐retest reliability.^33^ Content validity was assessed by the item‐level content validity index (I‐CVI) and the scale‐level content validity index (S‐CVI). CVIs were assessed by 3 rheumatologists as experts. Each expert rated each item either 1 (not relevant), 2 (somewhat relevant), 3 (quite relevant) or 4 (highly relevant). Then, for each item, the I‐CVI was computed as the number of experts giving a rating of either 3 or 4 (thus dichotomizing the ordinal scale into relevant and not relevant), divided by the total number of experts. The S‐CVI was measured by averaging calculation method (S‐CVI/Ave), that is, by the average of the I‐CVIs for all items on the scale. The scale was judged to have excellent content validity if the I‐CVI = 1 for each item and the S‐CVI/Ave ≥ 0.9, as recommended by Polit and Beck (2006).^34^

The Mann–Whitney *U* test (also called the Mann–Whitney–Wilcoxon (MWW), Wilcoxon rank‐sum test, or Wilcoxon–Mann–Whitney test) was used to compare between 2 groups with respect to a variable that does not follow a normal distribution.

The Kruskal‐Wallis test (sometimes also called the "one‐way analysis of variance on ranks") is a rank‐based nonparametric test. It was used to determine whether there are statistically significant differences between 2 or more groups of an independent variable on a continuous or ordinal dependent variable.

The ICC or the intraclass correlation, is a descriptive statistic. It was used when quantitative measurements are made on units that are organized into groups. It describes how strongly units in the same group resemble each other.

### 2.7 Ethical clearance

The Institutional Review Board (IRB) of Bangabandhu Sheikh Mujib Medical University provided clearance to conduct the study (No. BSMMU IRB 11606). All the participants were informed in details about the nature of the study. Only the individuals willing to participate in the study were included. Informed written consent was taken from the participants. Every participant enjoyed his/her right to participate or refuse to participate and to withdraw participation at any time. The principal investigator maintained the confidentiality of the information obtained from the participants. Data were intended to be used solely for this study.

## 3 RESULTS

### 3.1 Socio‐demographic data

A total of 95 patients could be included in the study. Their mean age was 37 years (SD 13.01), 43 male (45.3%) and 52 female (54.7%). The rheumatological diagnoses are summarized in Table 1. There were 27 patients (24.8%) who were below the secondary education level and 68 patients (71.6%) were at secondary level and above (Table 1). Thirty‐eight patients (40%) came from a rural area and 57 (60%) patients were from the urban area. We found no significant difference between patients BePCS scores and their age (*P* = .971), gender (Table 2) or educational level (*P* = .145). Although BePCS scores were lower in people with higher educational levels, the differences were insignificant as per the Kruskal‐Wallis test (Table 3). The BePCS total and subscale scores were higher in females but this difference was not statistically significant (Table 2).

**Table 1:** Socio‐demographic profile of the respondents

Characteristics	Frequency (n)	Percentage (%)
Age, y		
≤20	7	7.4
21‐30	27	28.4
31‐40	24	25.3
41‐50	23	24.2
>50	14	14.7
Gender		
Male	43	45.3
Female	52	54.7
Residence		
Rural	38	40.0
Urban	57	60.0
Educational status		
Below primary	13	13.7
Primary level	14	14.7
Secondary level	27	28.4
Higher secondary level	13	13.7
Bachelor/Master degree	28	29.5
Occupation		
Housewife	37	38.9
Govt. service	6	6.3
Private service	11	11.6
Businessman	15	15.8
Farmer	3	3.2
Rickshaw or van puller	4	4.2
Student	19	20.0
Disorders		
Rheumatoid arthritis	35	36.84
Spondyloarthritis (peripheral)	21	22.1
Chronic mechanical back pain	12	12.63
Ankylosing spondylitis	11	11.57
Psoriatic arthritis	09	9.47
Fibromyalgia	4	4.21
Systemic lupus erythematosus	3	3.15

**Table 2:** Total and subscale scores (medians) as per the Bengali Pain Catastrophizing Scale in male and female patients with chronic non‐malignant pain

Variable	Median (all participants)	Median (male)	Median (female)	*P* value*
Rumination subscale	9	9	9	.460
Magnification subscale	4	3	4	.337
Helplessness subscale	6	6	8	.289
PCS total	21	19	22.5	.257

* Mann‐Whitney *U* test.

**Table 3:** Relation between educational level and catastrophizing as measured by Bengali Pain Catastrophizing Scale (PCS)

Educational status	PCS (median)	*P*
Illiterate	24	.145*
Capable of reading only	29	
Capable of both reading and writing	35	
Primary level	25	
Secondary level	16	
Higher secondary level	17	
Bachelor/Master degree	16	

* Kruskal‐Wallis test.

### 3.2 Content validity

The I‐CVI and the S‐CVI were the assessment tools of content validity. All items of the scale showed excellent content validity: both I‐CVI and S‐CVI were 1.

### 3.3 Construct validity

Convergent validity was measured by comparing the BePCS score with the scores of the physical functioning and mental health domain of the Bengali version of the SF‐36^30^ through Spearman's correlation coefficient (rS). The convergent validity was found to be −0.424 for physical functioning and −0.413 for mental health, indicating a moderate negative correlation.

### 3.4 Internal consistency and test‐retest reliability

The total BePCS score showed an excellent total internal consistency with Cronbach's alpha of 0.92. Internal consistency for subscales rumination, magnification and helplessness, were Cronbach's *α* 0.903, 0.72 and 0.872, respectively (Table 4).

**Table 4:** Internal consistency of subgroups of the Bengali Pain Catastrophizing Scale (PCS)

Cronbach's *α*	PCS subscales	No. of items
0.903	Rumination	4
0.72	Magnification	3
0.872	Helplessness	6
0.92	Total PCS	13

The test‐retest reliability of the BePCS scale was measured by the ICC. The test‐retest reliability of the total BePCS was 0.781 (*P* < .001) and for the subscales rumination 0.872 (*P* < .001), magnification 797 (*P* < .001) and helplessness 0.927 (*P* < .001), indicative of a strong correlation between test and retest scores and hence showing excellent test‐retest reliability (Table 5).

**Table 5:** Correlation coefficients (*r*) between Bengali Pain Catastrophizing Scale (PCS) and subscales

	*r*	*P*
Rumination	.872	<.001
Magnification	.797	<.001
Helplessness	.927	<.001
Total PCS	.781	<.001

## 4 DISCUSSION

The pivotal components of cultural adaptation of a standard scale or instrument are translation and standardization of questionnaires. Occasionally assembling appropriate words pose a great challenge for translators. The PCS is the most commonly used tool to assess catastrophizing patients suffering from chronic pain. This study intended to validate the culturally adapted Bengali version of the PCS in adult Bangladeshi patients suffering from chronic non‐malignant musculoskeletal pain. The process of translating and back‐translating the English PCS to BePCS was carried out in accordance with the established guideline of Beaton et al^29^ After the validation of the original scale, all three subscales of the Bengali version (rumination, magnification, and helplessness), as well as the total of the scale, showed good internal consistency and similar correlation coefficients with the original scale except magnification subscale. The Cronbach's *α* of our study were 0.90, 0.72, 0.87 and 0.92 for the subscales rumination, magnification and helplessness and the total PCS scale respectively, comparable with those reported in the original study of Sullivan^19^ where the values were 0.87, 0.66, 0.78 and 0.87 respectively. The internal consistency for helplessness, magnification, rumination, and total scale of the Korean PCS was Cronbach's *α* = 0.90, 0.71, 0.86, and 0.93 respectively^14^ which is consistent with our study. Another study conducted by Suren et al^10^ also found a low Cronbach's *α* of magnification subscale: 0.55. A possible explanation of the low Cronbach's *α* of the magnification subscale may be that it has only few items.^35^ Moreover, some of our patients got afraid listening to the statement of magnification subscale “I wonder whether something serious things may happen”.

We observed that PCS scores were nonsignificantly higher in female compared to male patients. The possible explanation may be our female population were more occupied with household activities individually and manually. Women experienced pain more intensely due to lower threshold to pressure pain than men.^10^ Fibromyalgia and attention seeking behavior from the family members or spouse may be a contributory factor besides the physical factors. Moreover in our study the highest number of patients were suffering from rheumatoid arthritis which is a female predominant disease. Studies conducted by Suren et al^10^ and Turner and Clancy^36^ showed higher PCS scores in females. But Granot and Ferber^37^ and Ruscheweyh et al^38^ ^,^ ^39^ reported that male and female patients did not significantly differ regarding the extent of pain catastrophizing.

The convergent validity was examined by investigating the relationship between BePCS scores and physical functioning and mental health domains of the SF‐36. The correlation coefficients for these relationships were −0.424 and −0.413, which means there were moderately negative correlations between the BePCS and physical and psychological functioning respectively. These results, in general, were consistent with other studies.^14^ Our finding that the PCS scores correlated negatively more with physical than with psychological functioning may be explained by the fact that in the other studies the participants were collected from pain clinics where headache and other types of functional pain are seen more often,^23^, ^24^ whereas, our study predominantly included patients with rheumatoid arthritis, spondyloarthritis and ankylosing spondylitis (Table 1).

The test‐retest reliability of the BePCS showed excellent ICC of 0.78 which was consistent with the study conducted by Cho, Kim, and Lee^14^ (ICC = 0.79) and with the original English version of PCS (ICC = 0.75) by Sullivan.^19^

Age is another factor evaluated in studies associated with PCS scores.^37^, ^38^ ^,^ ^39^ They did not find any correlation between age and the PCS score. In the present study also no significant correlation was found between age and the total PCS or PCS subscale scores. In our study, we looked for a possible relation between educational level and catastrophizing; the PCS scores of the lower literacy group were higher than those of the higher literacy group, but this was not statistically significant. Other studies described by Yap et al^22^ in China and Granot and Ferber^36^ in a group of 38 Israeli patients also found no impact of educational level with PCS scores. Suren et al^9^ on the other hand found that PCS scores of high school graduates in Turkey were higher than those of primary school graduates. Further studies in other countries are needed regarding the relationship between PCS scores and educational status.

Pain catastrophizing has a social function and could affect family or significant others. It has been found in some previous studies that patients having higher PCS scores consumed higher amounts of analgesics and suffered from chronic and severe pain.^40^, ^41^ That is why the PCS has been developed into several other versions.^42^

### 4.1 Limitations

Our study showed some limitations. We could not study a possible correlation between various psychological scores, pain and disability (eg, Beck Depression Inventory, Pain Anxiety Symptom Scale‐20 etc) as was done in some other studies (eg, Korean PCS)^13^ as these scales have not yet been validated in Bengali. As our study was carried out in a tertiary level hospital, it may not be fully representative for the whole Bengali speaking population. Sensitivity to change could not be evaluated due to temporal constraint.

A strength of the study is that it is the first study in the Bengal language and it will create opportunities to study this important field of catastrophizing and chronic pain in 265 million Bengali speaking people. Our study showed acceptable validity and excellent internal consistency, construct and content validity and reliability of the Bengali version of the PCS.

In conclusion, the BePCS, being a valid and reliable tool, may be used to screen the probability of catastrophizing when suffering from chronic pain. The BePCS can be a valuable tool for patient education, treatment planning and to assess the need for psychological intervention.

## Supporting information

Bengali PCS

## References

[1] Morone NE , Weiner DK . Pain as the fifth vital sign: exposing the vital need for pain education. Clin Ther. 2013;35(11):1728‐1732. 24145043 10.1016/j.clinthera.2013.10.001 PMC3888154

[2] Gatchel RJ , Peng YB , Peters ML , Fuchs PN , Turk DC . The biopsychosocial approach to chronic pain: scientific advances and future directions. Psychol Bull. 2007;133(4):581‐624. 17592957 10.1037/0033-2909.133.4.581

[3] Klaber Moffett J , Richardson G , Sheldon TA , Maynard A . Back Pain. Its Management and Cost to Society (Discussion paper No 129.) University of York, Centre for Health Economics, NHS Centre for Reviews and Dissemination. A short Guide to Purchasers on the State of the Evidence for Primary Care Management of Acute and Chronic Back Pain Nuffield Institute for Health, Leeds; 1995.

[4] Frymoyer JW , Ducker TB , Hadler NM , Kostuik JP . The Adult Spine: Principles and Practice. Philadelphia, PA: Lippincott‐Raven Publishers; 1997.

[5] Nachemson AL , Waddell G , Norlund A . Epidemiology of neck and low back pain In: Nachemson AL, Jonsson E, eds. Neck and Back Pain: The Scientific Evidence of Causes, Diagnosis and Treatment. Philadelphia, PA: Lippincott Williams & Wilkins; 2000.

[6] Woolf AD , Akesson K . Understanding the burden of musculoskeletal conditions. The burden is huge and not reflected in national health priorities. BMJ. 2001;322(7294):1079‐1080. 11337425 10.1136/bmj.322.7294.1079 PMC1120225

[7] Meyer K , Tschopp A , Sprott H , Mannion AF . Association between catastrophizing and self‐rated pain and disability in patients with chronic low back pain. J Rehabil Med. 2009;41(8):620‐625. 19565155 10.2340/16501977-0395

[8] Suso‐Ribera C , Garcia‐Palacios A , Botella C , Ribera‐Canudas MV . Pain catastrophizing and its relationship with health outcomes: does pain intensity matter? Pain Res Manag. 2017;2017:9762864. 28348506 10.1155/2017/9762864 PMC5350380

[9] Darnall BD , Sturgeon JA , Cook KF , et al. Development and validation of a daily pain catastrophizing scale. J Pain. 2017;18(9):1139‐1149. 28528981 10.1016/j.jpain.2017.05.003 PMC5581222

[10] Süren M , Okan İ , Gökbakan AM , et al. Factors associated with the pain catastrophizing scale and validation in a sample of the Turkish population. Turk J Med Sci. 2014;44(1):104‐108. 25558568 10.3906/sag-1206-67

[11] Edwards RR , Bingham CO III , Bathon J , Haythornthwaite JA . Catastrophizing and pain in arthritis, fibromyalgia, and other rheumatic diseases. Arthritis Rheum. 2006;55(2):325‐332. 16583384 10.1002/art.21865

[12] Sullivan MJ , Adams H , Sullivan ME . Communicative dimensions of pain catastrophizing: social cueing effects on pain behaviour and coping. Pain. 2004;107(3):220‐226. 14736584 10.1016/j.pain.2003.11.003

[13] Turk DC , Rudy TE . Cognitive factors and persistent pain: a glimpse into Pandora's box. Cogn Ther Res. 1992;16(2):99‐122.

[14] Cho S , Kim HY , Lee JH . Validation of the Korean version of the Pain Catastrophizing Scale in patients with chronic non‐cancer pain. Qual Life Res. 2013;22(7):1767‐1772. 23180163 10.1007/s11136-012-0308-2

[15] Raeissadat SA , Sadeghi S , Montazeri A . Validation of the pain catastrophizing scale (PCS) in Iran. J Basic Appl Sci Res. 2013;3:376‐380.

[16] Gibson E , Sabo MT . Can pain catastrophizing be changed in surgical patients? A scoping review. Can J Surg. 2018;61:311‐318. 30246983 10.1503/cjs.015417 PMC6153100

[17] Sullivan MJ , Martel MO , Tripp D , Savard A , Crombez G . The relation between catastrophizing and the communication of pain experience. Pain. 2006;122(3):282‐288. 16545907 10.1016/j.pain.2006.02.001

[18] Sullivan MJ , Martel MO , Tripp DA , Savard A , Crombez G . Catastrophic thinking and heightened perception of pain in others. Pain. 2006;123(1–2):37‐44. 16563630 10.1016/j.pain.2006.02.007

[19] Sullivan MJL , Bishop SR , Pivik J . The pain catastrophizing scale: development and validation. Psychol Assess. 1995;7(4):524‐532.

[20] Osman A , Barrios FX , Kopper BA , Hauptmann W , Jones J , O'Neill E . Factor structure, reliability, and validity of the Pain Catastrophizing Scale. J Behav Med. 1997;20(6):589‐605. 9429990 10.1023/a:1025570508954

[21] Cavalcante JA , Viana KA , Costa PS , Costa LR . Translation, cross‐cultural adaptation and preliminary evaluation of the Brazilian version of the pain catastrophizing scale‐parents. Rev Paul Pediatr. 2018;36(4):428‐436. 30540108 10.1590/1984-0462/;2018;36;4;00014 PMC6322810

[22] Sehn F , Chachamovich E , Vidor LP , et al. Cross‐cultural adaptation and validation of the Brazilian Portuguese version of the pain catastrophizing scale. Pain Med. 2012;13(11):1425‐1435. 23036076 10.1111/j.1526-4637.2012.01492.x

[23] Yap JC , Lau J , Chen PP , et al. Validation of the Chinese Pain Catastrophizing Scale (HK‐PCS) in patients with chronic pain. Pain Med. 2008;9(2):186‐195. 18298701 10.1111/j.1526-4637.2007.00307.x

[24] Terkawi AS , Sullivan M , Abolkhair A , et al. Development and validation of Arabic version of the pain catastrophizing scale. Saudi J Anaesth. 2017;11(Suppl 1):S63. 28616005 10.4103/sja.SJA_130_17 PMC5463568

[25] Population Bangladesh . (2020–02‐17). Retrieved 2020–03‐23, http://worldpopulationreview.com/countries/bangladesh/. Accessed March 15, 2020.

[26] Eberhand DM , Simons GF , and Fenning CD (eds.). 2020 Ethnologue: Languages of the World, 23rd edn Dallas, TX: SIL International http://www.ethnologue.com/statistics/size. Accessed May 2020.

[27] http://www.studysize.com.

[28] Sullivan MJL . PCS: The Pain Catastrophizing Scale (User Manual). Montreal, Canada: Publication of McGill University; 2009 Page 4. http://sullivanpainresearch.mcgill.ca/pdf/pcs/PCSManual_English.pdf. Accessed June 2015.

[29] Beaton DE , Bombardier C , Guillemin F , Ferraz MB . Guidelines for the process of cross‐cultural adaptation of self‐report measures. Spine (Phila PA, 1976). 2000;25(24):3186‐3191. 11124735 10.1097/00007632-200012150-00014

[30] Feroz AH , Islam MN , ten Klooster PM , Hasan M , Rasker JJ , Haq S . The Bengali Short Form‐36 was acceptable, reliable, and valid in patients with rheumatoid arthritis. J Clin Epidemiol. 2012;65:1227‐1235. 23017640 10.1016/j.jclinepi.2012.05.004

[31] George D , Mallery P . SPSS for Windows Step by Step: A Simple Guide and Reference 11.0 Update, 4th edn Boston: Allyn & Bacon; 2003.

[32] Baum CF 2005 SPEARMAN2: Stata Module to Calculate Spearman Rank Correlations, Extended. Boston, MA: Statistical Software Components S454301, Boston College Department of Economics.

[33] Cicchetti DV . Guidelines, criteria, and rules of thumb for evaluating normed and standardized assessment instruments in psychology. Psychol Assess. 1994;6:284‐290.

[34] Polit DF , Beck CT . The content validity index: are you sure you know what's being reported? Critique and recommendations. Res Nurs Health. 2006;29(5):489‐497. 16977646 10.1002/nur.20147

[35] Osburn HG . Coefficient alpha and related internal consistency reliability coefficients. Psychol Methods. 2000;5(3):343‐355. 11004872 10.1037/1082-989x.5.3.343

[36] Turner JA , Clancy S . Strategies for coping with chronic low back pain: relationship to pain and disability. Pain. 1986;24(3):355‐364. 2938059 10.1016/0304-3959(86)90121-1

[37] Granot M , Ferber SG . The roles of pain catastrophizing and anxiety in the prediction of postoperative pain intensity: a prospective study. Clin J Pain. 2005;21(5):439‐445. 16093750 10.1097/01.ajp.0000135236.12705.2d

[38] Papaioannou M , Skapinakis P , Damigos D , Mavreas V , Broumas G , Palgimesi A . The role of catastrophizing in the prediction of postoperative pain. Pain Med. 2009;10(8):1452‐1459. 19863742 10.1111/j.1526-4637.2009.00730.x

[39] Ruscheweyh R , Nees F , Marziniak M , Evers S , Flor H , Knecht S . Pain catastrophizing and pain‐related emotions: influence of age and type of pain. Clin J Pain. 2011;27(7):578‐586. 21368662 10.1097/AJP.0b013e31820fde1b

[40] Sullivan MJL , Thorn B , Haythornthwaite JA , et al. Theoretical perspectives on the relation between catastrophizing and pain. Clin J Pain. 2001;17(1):52‐64. 11289089 10.1097/00002508-200103000-00008

[41] Keefe FJ , Rumble ME , Scipio CD , Giordano LA , Perri LM . Psychological aspects of persistent pain: current state of the science. J Pain. 2004;5(4):195‐211. 15162342 10.1016/j.jpain.2004.02.576

[42] Cano A , Leonard MT , Franz A . The significant other version of the Pain Catastrophizing Scale (PCS‐S): preliminary validation. Pain. 2005;119(1–3):26‐37. 16298062 10.1016/j.pain.2005.09.009 PMC2679670

