# Supplementary material for: Translation, cross‐cultural adaptation and validation of the Pain Catastrophizing Scale (PCS) into Bengali in patients with chronic non‐malignant musculoskeletal pain
Source: Int J Rheum Dis. 2020 Aug 30;23(11):1481–7. doi: 10.1111/1756-185X.13954 (PMC7754436; doi:10.1111/1756-185X.13954)
Supplement: Supplementary file 1 — Bengali PCS [file APL-23-1481-s001.docx]

**Adapted Bengali version of PCS**

| **Pain catastrophizing scale** | **Range** |
| --- | --- |
| **Rumination subscale (‡ivg¯’b Dc-gvb`Ð)** | 0-16 |
| I anxiously want the pain to go away.  8. Avwg gb †_‡K PvB e¨_vUv P‡j hvK \| | 0-4 |
| I can’t seem to keep it out mind.  9. Avwg †Kvb fv‡e e¨_vUv fz‡j _vK‡Z cviwQ bv\| | 0-4 |
| I keep thinking about how much it hurts.  10. GwU †h ‡Kgb Kó †`q Zv Avwg memgq wPšÍv Ki‡Z _vwK\| | 0-4 |
| I keep thinking about how badly I want the pain to stop.  11. e¨_v †_‡g hvK-G wb‡q Avwg †h KZUv wPwšÍZ Zv Avwg fve‡Z _vwK \| | 0-4 |
| **Magnification subscale ( AwZiÄb Dc-gvb`Ð)** | 0-12 |
| I become afraid that the pain may get worse.  6. Avwg f‡q _vwK Avgvi e¨_vUv Av‡iv Lvivc n‡Z cv‡i\| | 0-4 |
| I think of other painful experiences.  7. Ab¨vb¨ †e`bv`vqK NUbvi K_v Avgvi g‡b c‡o \| | 0-4 |

| I wonder whether something serious may happen.  13. Avgvi g‡b nq gvivZ¥K wKQz NU‡Z cv‡i\| | 0-4 |
| --- | --- |
| **Helplessness subscale ( AmnvqZ¡ Dc-gvb`Ð)** | 0-24 |
| I worry all the time whether the pain will end.  1. KL‡bv G e¨_v †kl n‡e wK bv Zv wb‡q Avwg wPwšÍZ _vwK \| | 0-4 |
| I feel I can’t go on.  2. Avgvi g‡b nq Avwg Gfv‡e †ewkw`b Pj‡Z cvi‡ev bv\| | 0-4 |
| It’s terrible and I think it’s never going to get. any better  3. GUv fq¼i, Avgvi g‡b nq G †_‡K Avwg KL‡bv gyw³ cv‡ev bv \| | 0-4 |
| It’s awful and I feel that it overwhelms me.  4. GUv fqven, Avgvi g‡b nq GUv Avgv‡K ‡kl K‡i w`‡”Q \| | 0-4 |
| I feel I can’t stand it anymore.  5. Avgvi g‡b nq Avwg Avi mn¨ Ki‡Z cviwQ bv \| | 0-4 |
| There is nothing I can do to reduce the intensity of the pain.  12. e¨_vi gvÎv Kgv‡bvi Rb¨ Avgvi Avi wKQzB Kivi †bB\| | 0-4 |
